# Prevalence of *Tritrichomonas foetus* Among Cats in Poland Between 2020 and 2024

**DOI:** 10.3390/pathogens14050458

**Published:** 2025-05-07

**Authors:** Dawid Jańczak, Klaudiusz Szczepaniak, Jagoda Jeleniewska, Olga Szaluś-Jordanow

**Affiliations:** 1 Department of Infectious and Invasive Diseases and Veterinary Administration, Institute of Veterinary Medicine, Faculty of Biological and Veterinary Sciences, Nicolaus Copernicus University, Gagrina 7, 87-100 Toruń, Poland; 2 Department of Parasitology and Fish Diseases, Faculty of Veterinary Medicine, University of Life Sciences in Lublin, Akademicka 12, 20-033 Lublin, Poland; 3 Department of Public Health Protection and Animal Welfare, Institute of Veterinary Medicine, Faculty of Biological and Veterinary Sciences, Nicolaus Copernicus University, Gagarina 7, 87-100 Toruń, Poland; jagoda.jeleniewska@umk.pl; 4 Department of Small Animal Diseases with Clinic, Institute of Veterinary Medicine, Warsaw University of Life Sciences-SGGW, Nowoursynowska 159c, 02-776 Warsaw, Poland; olga_szalus-jordanow@sggw.edu.pl

**Keywords:** *Tritrichomonas foetus*, feline trichomonosis, cats, large-bowel diarrhea, nested PCR, Poland

## Abstract

*Tritrichomonas foetus* is a parasitic protozoan that causes large-bowel disorders in domestic cats worldwide. Between 2020 and 2024, 1606 feline stool samples were tested for *T. foetus*. The aim of this study was to determine the prevalence of *T. foetus* infection among cats with diarrhea reported within the last 6 months, and to assess age and sex as risk factors for *T. foetus* infection. The diagnostic method of choice was nested PCR with modifications to reduce potential reaction inhibition. Overall, *T. foetus* DNA was detected in 10.6% (170/1606) of cats, with the highest prevalence observed in animals younger than one year, 14.9% (106/711). This study confirms that *T. foetus* infection is common in domestic cats in Poland. Therefore, this parasite should be included in the differential diagnosis protocol for feline patients with large-bowel diarrhea.

## 1. Introduction

*Tritrichomonas foetus* is a single-celled, flagellated protozoan belonging to the phylum Parabasalia, order Trichomonadida, and Tritrichomonadidae family. The final name of the parasite was established by Wenrich and Emmerson [1,2]. It has been recognized as a pathogenic protozoan of cattle and cats, and possibly as a commensal in pigs [3]. However, molecular analyses confirm that pigs can be infected by both the “cat-genotype” and “cattle-genotype” of *T. foetus* [4]. In cattle, *T. foetus* is a sexually transmitted disease that causes early foetus abortion [5]. The parasite can be found on the mucous membrane of the urogenital tract, where it induces inflammation of the uterus and vagina [6,7,8]. In cats, *T. foetus* is responsible for chronic, large intestinal disorders, particularly in young felines. Initially, the primary trichomonad causing diarrhea in domestic cats was identified as *Pentatrichomonas hominis* [9]. However, in 2001, the parasite was subsequently confirmed as *T. foetus* [10]. Notably, flagellate protozoan infection in cats has already been observed as early as the 1930s [11,12]. Feline infection of *T. foetus* is characterized by recurrent, chronic diarrhea with loose, cow-pie-like feces containing fresh blood and mucus [9,13]. The parasite colonizes the distal ileum and colon, where it induces neutrophilic and lymphoplasmacytic colitis in both clinically and experimentally infected cats [14]. Transmission between cats occurs via the fecal-oral route. In moist stool, *T. foetus* trophozoites remain viable and infective for several days [15]. Trichomonosis affects cats of all ages but is more common in young cats at approximately one year old [3,16,17]. Another significant risk is the high-density living conditions and close contact between cats, which facilitate the fecal-oral transmission of the parasite [3]. Consequently, catteries have been identified as a location with high infection rates [16]. Additionally, the odds ratio for infection is higher in purebred cats compared to non-purebred cats [3,18]. Chronic diarrhea can persist for up to two years, but asymptomatic infection can last for life [9,19,20]. Diagnosing *T. foetus* infection in cats can be performed using direct smear examination, fecal culture, polymerase chain reaction (PCR), and colonic mucosal biopsy. Fecal samples for microscopic examination can be obtained via enema, per rectum fecal loop, or by collecting fresh, moist stool samples from cat litter [9,19]. Only fresh diarrheic stool samples are suitable for direct smear examination within six hours of excretion. However, this method has the lowest sensitivity [21]. Fecal culture in Modified Detmer’s Medium (MDM) (26.4% sensitivity) or the commercially available InPouch™ TF medium (58.8% sensitivity) (InPouch TF; BioMed Diagnostics, Inc., White City, OR, USA) is more sensitive than the direct fecal smear (14.7% sensitivity) [15,22,23]. PCR, which amplifies the first (ITS1) and second (ITS2) internal transcribed spacer regions and the 5.8S rRNA gene, is the most sensitive method for detecting *T. foetus* directly from stool samples. PCR can detect DNA from both live and dead organisms, with a detection sensitivity assessed at 10 *T. foetus* protozoa per 100 mg of feces [24,25,26]. Moreover, it is likely that the administration of antibacterial drugs and fecal samples collected simultaneously appears to reduce the chance of Tritrichomonas species detection [13]. Consequently, it is sensible to use molecular techniques in the diagnosis of feline Trichomonosis. *T. foetus* was detected for the first time in 2015 in Poland [27]. Since then, studies on the prevalence of this parasite in different groups of animals in Poland have been carried out. This manuscript focuses on the prevalence of *T. foetus* among domestic cats in Poland with a clinical history of diarrhea.

## 2. Materials and Methods

### 2.1. Samples Collection and DNA Extraction

Between 2020 and 2024, 1606 stool samples from cats with clinical suspicion of intestinal trichomonosis were collected in a commercial veterinary laboratory. Samples were submitted by veterinarians all around the country as part of the laboratory service tests. Stool samples were mechanically homogenized, and 0.5 g of stool was frozen at −25 °C for 24 h before DNA isolation. Following this pre-treatment, 100 mg of the sample was used for DNA extraction. The manual DNA extraction method was performed according to the manufacturer’s protocol (Genomic Mini AX Stool, A&A Biotechnology, Gdańsk, Poland). The extracted DNA was suspended in 200 μL of nuclease-free water and stored at 4 °C overnight. On the following day, Anti-Inhibitor Kit (A&A Biotechnology, Gdańsk, Poland) was used to eliminate PCR reaction inhibitors from suspended DNA. A total of 50 μL of purified DNA was frozen at −25 °C for further testing.

### 2.2. Nested PCR Detection Method

Nested PCR analysis was performed using a previously described protocol [24,25] with minor modifications, including two separate rounds of PCR amplification and dilution of first-round PCR products. The first-round reaction was carried out in a 50 μL reaction volume, which contained 25 μL StartWarm HS-PCR Mix A&A Biotechnology, Gdańsk, Poland), 2 μL of each primer TFR3 (5′-CGGGTCTTCCTATATGAGACAGAACC-3′) and TFR4 (5′-CCTGCCGTTGGATCAGTTTCGTTAA-3′) [24], 16 μL ddH2O, and 5 μL of purified genomic DNA. PCR was carried out by an initial denaturation at 95 °C for 3 min, followed by 45 cycles of denaturation at 95 °C for 30 s, annealing at 67 °C for 45 s, extension at 72 °C for 45 s, and a final extension at 72 °C for 10 min in a PCR thermocycler (MultiGene optiMAX, Labnet International, Inc., Taoyuan, Taiwan).

The second round was conducted in a 50 μL reaction volume, containing 2 μL each of primer TFITS-F (5′-CTGCCGTTGGATCAGTTTCG-3′) and TFITS-R (5′-GCAATGTGCATTCAAAGATCG-3′) [25] and 5 μL of 10-fold dilution of first-round PCR product. Reaction conditions were as follows: 3 min of initial denaturation at 95 °C, followed by 45 cycles at 95 °C for 30 s, annealing at 57 °C for 45 s, extension at 72 °C for 45 s, and final extension at 72 °C for 10 min. Both amplified products (347 bp and 208 bp) were analyzed on 2% agarose gel.

### 2.3. Statistical Analysis

The results were processed statistically in the Statistica 13.1 program (StatSoft medical application). The prevalence was expressed as a percentage of positive samples among the total tested stool samples. The presence of *T. foetus* was considered as a binary factor (POSITIVE/NEGATIVE reaction). To ensure the accuracy of this estimate, a ±95% confidence interval was calculated to ensure this estimate’s accuracy. The data were tested for normal distribution using the Kolmogorov–Smirnov test. To compare differences in prevalence, Student’s t-test and one-way ANOVA were applied to compare differences in prevalence. A *p*-value < 0.05 was considered statistically significant.

## 3. Results

Age information was available for 1421 (88.4%) cats, while sex data was recorded for 391 (24.3%) of them. Overall, *T. foetus* DNA was detected in 170 of the 1606 stool samples (10.6%), (95% CI, 9.1–12.1). The highest prevalence of *T. foetus* was observed among cats younger than 1 year of age (14.9%), (95% CI, 12.3–17.5). This age group showed a statistically significant difference compared to other age groups as well as to the overall prevalence (Table 1). Additionally, a decreasing trend in prevalence was observed with increasing age, with the lowest prevalence in the oldest age group (4.3%), (95% CI, 1.1–7.4). There was no significant difference based on sex (Table 2).

## 4. Discussion

*T. foetus* is an economically significant pathogen, causing early abortion and infertility in cattle [28]. In felines, infections associated with chronic large-bowel diarrhea have been widely reported, with a 10–59% prevalence in different regions worldwide [18,20,23,26,29,30,31,32,33,34,35,36,37,38,39]. Studies conducted in European countries report the prevalence of *T. foetus* infections among cats as follows: 14.4% (16/111) in the United Kingdom [16], 24.4% (11/45) in Switzerland [32], 32% (24/74) [17], 5.2% (14/267) [40] in Italy, 20.0% (6/30) in Greece [41], 25% (5/20) in Spain [42], 15.7% (36/230) in Germany [43], 21% (11/52) in Norway [44], 14.3% (20/140) in France [45], and 20.5% (24/117) in Poland [3].

In our study, the overall prevalence was 10.6% (170/1606), which was slightly lower than the prevalence determined by PCR in the United Kingdom, Germany, and France [16,43,45] but higher than in Italy [40]. A comparable study, a large animal group study conducted in the United States, analyzed 1717 samples, reporting a 16% prevalence among cats. The authors of the study found no significant association between age or sex and positive *T. foetus* [46], a conclusion similar to other studies [26,41]. Our findings confirm that, despite a selection bias towards symptomatic animals, the overall prevalence remained relatively low compared to some previous studies. This suggests that additional factors, such as geographical distribution, diagnostic techniques, and differences in cat populations, may influence prevalence rates.

Our findings confirm that cats aged one year or younger are at higher risk for *T. foetus* infection. In the present study, the mean and median ages of 1421 cats were 2.8 years and 1.1 years, respectively (range 1 month to 19.5 years). A significant difference was observed between cats aged one year or younger (106/711; 14.9%) and older cats (48/710; 6.7%), which aligns with previous studies [3,16,17,45]. This confirms that younger cats are at a higher risk of *T. foetus* infection. An exception to this trend was observed in an Italian study, where no significant difference was found between infected cats younger than one year (8/56) and one year or older (10/49) [47].

Sex does not appear to be a significant risk factor for *T. foetus* infection. Only one study has identified male sex as a risk factor in kittens [48], whereas most studies, including ours, found no significant difference between infected male and female cats [3,26,41].

Close contact between cats living in high-density environments has been confirmed as a potential risk factor for *T. foetus* infection [49]. Studies report higher prevalence rates in larger groups (>4 animals, 27.7%), compared to smaller groups (≤4 animals, 16.6%) [3]. Furthermore, cats from shelters or catteries have an increased likelihood of infection [16,45]. Our study did not analyze the impact of housing conditions on infection rates, which represents a limitation of our findings. Future research should focus on assessing the role of environmental factors and cat group sizes in disease transmission.

The primary reason for submitting feline fecal samples for PCR testing was a history of acute, intermittent, or chronic diarrhea within the last six months. The stool is often loose, yellowish, and malodorous [9,23]. In some kittens, fecal incontinence and anal inflammation may also develop [20]. Other clinical symptoms, such as vomiting, anorexia, weight loss, and depression, have been reported, though subclinical infection can also occur [9]. The association between diarrhea and *T. foetus* infection is well-documented. A study conducted in the USA has described a high prevalence of Trichomonosis with 22 out of 26 diarrheic cats (84.6%) testing positive [50]. *T. foetus* was confirmed in 24 out of 72 cats (33.3%) with large-bowel disorders through direct smears and in Pouch TF culture medium from rectal swabs [17]. Fecal samples collected via fecal loop were 2.04 times more likely to yield positive PCR results compared to the colonic flush technique [46].

No diagnostic test has 100% sensitivity. Molecular diagnostics, particularly PCR-based methods, have demonstrated the highest sensitivity (>90%) in detecting *T. foetus* from stool samples. In contrast, direct microscopy (14.7%) or culture technique (26.4% for MDM medium and 58.8% for In Pouch TF medium) are less effective [26,51]. Various PCR methods, including conventional PCR, nested PCR, real-time PCR, or Loop-mediated isothermal amplification (LAMP), differ in sensitivity and specificity [3,9,10,24,26,52]. The quality of DNA isolation is crucial for accurate PCR results, as inhibitors in stool samples (e.g., polysaccharides, bile salts, hemoglobin, urea, lipids, and polyphenols) can affect amplification efficiency [53,54]. Comparative studies evaluating different DNA extraction kits suggest that using 100 mg of stool improves DNA purity [55]. In our research, the Genomic Mini AX Stool (A&A Biotechnology, Gdańsk, Poland) kit was used along with two inhibition removal steps: the Anti-Inhibitor Kit (A&A Biotechnology, Gdańsk, Poland) and 10-fold dilution of first-round PCR products before nested PCR. The DNA isolation was performed using 100 mg of feces, as recommended by the manufacturer’s protocol.

Nested PCR was chosen due to its higher sensitivity compared to single-round PCR reactions (90% vs. 30–70%) and the ability to detect as few as 10 *T. foetus* organisms per 100 mg of feces [55]. A Polish study comparing conventional PCR (17.9%, 21/117) and (LAMP) (20.5%, 24/117) further supports the importance of molecular assays for accurate detection [3]. The MT-PCR and TaqMan-MGB real-time PCR assays were evaluated. The MT-PCR panel demonstrated high sensitivity and specificity for *Giardia intestinalis* (95.1%; 92.1%) but lower sensitivity for *T. foetus* (41.9%). This difference was attributed to target gene selection—ITS-1 (multi-copy) in TaqMan-MGB PCR versus the single-copy cysteine protease gene in MT-PCR [52]. Nonetheless, PCR assays targeting the internal transcribed spacer (ITS1, ITS2) and 5.8S rRNA genes remain widely used for *T. foetus* molecular diagnostics [3,9,10,24,25,26,52]. The choice of DNA isolation kit and suitable PCR appear to be important for reliable assessment of the prevalence among cat populations. Increased shedding of *T. foetus* in feces can be a consequence of antibiotic therapy, which can kill natural intestinal microbiota [56]. Our study provides valuable epidemiological data on the prevalence of *T. foetus* in cats, confirming that young age is a significant risk factor. Although our prevalence rate was lower than in some previous studies, nested PCR allowed for highly sensitive detection, according to the literature [25,55]. Our findings emphasize the need for standardized molecular diagnostics and further investigation into environmental risk factors. Future studies should explore the role of asymptomatic carriers and assess infection dynamics in multi-cat households.

## 5. Conclusions

This study confirms that *T. foetus* is commonly detected in cats with diarrhea. This protozoan parasite should be considered an essential differential diagnosis in feline large-bowel disorders, especially in kittens and young cats. As with other parasitic and infectious agents, *T. foetus* should be ruled out early in the diagnostic process for cats with chronic gastrointestinal signs.

## Figures and Tables

**Table 1:** Prevalence of *T. foetus* in different age groups.

Age	N	Positive	Prevalence (%)	±95% CI
overall	1606	170	10.6	9.1–12.1
≤1	711	106	14.9	12.3–17.5
>1–3	353	31	8.8	5.8–11.7
>3–7	194	10	5.2	2.0–8.3
>7–20	163	7	4.3	1.1–7.4
no data	185	16	8.6	4.6–12.7

**Table 2:** Prevalence of *T. foetus* depending on the sex of the tested cats.

Sex	N	Positive	Prevalence (%)	95% CI
male	104	17	16.3	9.1–23.6
female	287	41	14.3	10.2–18.4

## Data Availability

The original contributions presented in this study are included in the article. Further inquiries can be directed to the corresponding author.

## References

[1] Morgan B.B. (1947). A summary of research on *Trichomonas foetus*. J. Parasitol..

[2] Adl S.M., Simpson A.G., Farmer M.A., Andersen R.A., Anderson O.R., Barta J.R., Bowser S.S., Brugerolle G., Fensome R.A., Fredericq S. (2005). The new higher-level classification of eukaryotes with emphasis on the taxonomy of protists. J. Eukaryot. Microbiol..

[3] Dąbrowska J., Karamon J., Kochanowski M., Sroka J., Skrzypek K., Zdybel J., Różycki M., Jabłoński A., Cencek T. (2020). *Tritrichomonas foetus*: A Study of Prevalence in Animal Hosts in Poland. Pathogens.

[4] Doi J., Abe N., Oku Y. (2013). Molecular survey of Tritrichomonas suis (=*T. foetus*) ‘cat’ and ‘cattle’ genotypes in pigs in Japan. J. Vet. Med. Sci..

[5] Felleisen R.S. (1999). Host-parasite interaction in bovine infection with *Tritrichomonas foetus*. Microbes Infect..

[6] Yule A., Skirrow S.Z., Bonduran R.H. (1989). Bovine trichomoniasis. Parasitol. Today.

[7] Michi A.N., Favetto P.H., Kastelic J., Cobo E.R. (2016). A review of sexually transmitted bovine trichomoniasis and campylobacteriosis affecting cattle reproductive health. Theriogenology.

[8] Dąbrowska J., Karamon J., Kochanowski M., Sroka J., Zdybel J., Cencek T. (2019). *Tritrichomonas foetus* as a causative agent of tritrichomonosis in different animal hosts. J. Vet. Res..

[9] Gookin J.L., Breitschwerdt E.B., Levy M.G., Gager R.B., Benrud J.G. (1999). Diarrhea associated with trichomonosis in cats. J. Am. Vet. Med. Assoc..

[10] Levy M.G., Gookin J.L., Poore M., Birkenheuer A.J., Dykstra M.J., Litaker R.W. (2003). *Tritrichomonas foetus* and not *Pentatrichomonas* hominis is the etiologic agent of feline trichomonal diarrhea. J. Parasitol..

[11] Da Cuncha A.M., Muniz J. (1922). Trabalhos do instituto Oswaldo Cruz: Sobre um flagellado parasito gato. Braz. Med..

[12] Brumpt E. (1925). Recherches morphologiques et experimentales sur le Trichomonas felis da cunha et muniz, 1922, parasites do chat et du chien. Ann. Parasitol. Hum. Comp..

[13] Gookin J.L., Hanrahan K., Levy M.G. (2017). The condundrum of feline trichomonosis—The more we learn the “trickier” it gets. J. Feline Med. Surg..

[14] Yaeger M.J., Gookin J.L. (2005). Histologic features associated with *Tritrichomonas foetus*-induced colitis in domestic cats. Vet. Pathol..

[15] Gookin J.L., Foster D.M., Poore M.F., Stebbins M.E., Levy M.G. (2003). Use of a commercially available culture system for diagnosis of *Tritrichomonas foetus* infection in cats. J. Am. Vet. Med. Assoc..

[16] Gunn-Moore D.A., McCann T.M., Reed N., Simpson K.E., Tennant B. (2007). Prevalence of *Tritrichomonas foetus* infection in cats with diarrhoea in the UK. J. Feline Med. Surg..

[17] Holliday M., Deni D., Gunn-Moore D.A. (2009). *Tritrichomonas foetus* infection in cats with diarrhoea in a rescue colony in Italy. J. Feline Med. Surg..

[18] Hosein A., Kruth S.A., Pearl D.L., Richardson D., Maggs J.C., Peach H.A., Peregrine A.S. (2013). Isolation of *Tritrichomonas foetus* from cats sampled at a cat clinic, cat shows and a humane society in southern Ontario. J. Feline Med. Surg..

[19] Foster D.M., Gookin J.L., Poore M.F., Stebbins M.E., Levy M.G. (2004). Outcome of cats with diarrhea and *Tritrichomonas foetus* infection. J. Am. Vet. Med. Assoc..

[20] Stockdale H.D., Givens M.D., Dykstra C.C., Blagburn B.L. (2009). *Tritrichomonas foetus* infections in surveyed pet cats. Vet. Parasitol..

[21] Hale S., Norris J.M., Slapeta J. (2009). Prolonged resilience of *Tritrichomonas foetus* in cat feces at ambient temperature. Vet. Parasitol..

[22] Watanabe A. (1960). List of algal strains in collection at the Institute of Applied Microbiology, University of Tokyo. J. Gen. Appl. Microbiol..

[23] Yao C., Köster L.S. (2015). *Tritrichomonas foetus* infection, a cause of chronic diarrhea in the domestic cat. Vet. Res..

[24] Felleisen R.S., Lambelet N., Bachmann P., Nicolet J., Müller N., Gottstein B. (1998). Detection of *Tritrichomonas foetus* by PCR and DNA enzyme immunoassay based on rRNA gene unit sequences. J. Clin. Microbiol..

[25] Gookin J.L., Birkenheuer A.J., Breitschwerdt E.B., Levy M.G. (2002). Single-tube nested PCR for detection of *Tritrichomonas foetus* in feline feces. J. Clin. Microbiol..

[26] Gookin J.L., Stebbins M.E., Hunt E., Burlone K., Fulton M., Hochel R., Talaat M., Poore M., Levy M.G. (2004). Prevalence of and risk factors for feline *Tritrichomonas foetus* and giardia infection. J. Clin. Microbiol..

[27] Dąbrowska J., Karamon J., Kochanowski M., Jędryczko R., Cencek T. (2015). *Tritrichomonas foetus* infection in cat—First detection in Poland. Acta Parasitol..

[28] Yao C. (2021). Control and eradication of bovine trichomonosis in Wyoming, USA by testing and culling positive bulls. Vet. Res..

[29] Bissett S.A., Gowan R.A., O’Brien C.R., Stone M.R., Gookin J.L. (2008). Feline diarrhoea associated with Tritrichomonas cf. foetus and Giardia co-infection in an Australian cattery. Aust. Vet. J..

[30] Bissett S.A., Stone M.L., Malik R., Norris J.M., O’Brien C., Mansfield C.S., Nicholls J.M., Griffin A., Gookin J.L. (2009). Observed occurrence of *Tritrichomonas foetus* and other enteric parasites in Australian cattery and shelter cats. J. Feline Med. Surg..

[31] Burgener I., Frey C., Kook P., Gottstein B. (2009). *Tritrichomonas fetus*: A new intestinal parasite in Swiss cats. Schweiz. Arch. Tierheilkd..

[32] Frey C.F., Schild M., Hemphill A., Stünzi P., Müller N., Gottstein B., Burgener I.A. (2009). Intestinal *Tritrichomonas foetus* infection in cats in Switzerland detected by in vitro cultivation and PCR. Parasitol. Res..

[33] Bell E.T., Gowan R.A., Lingard A.E., McCoy R.J., Slapeta J., Malik R. (2010). Naturally occurring *Tritrichomonas foetus* infections in Australian cats: 38 cases. J. Feline Med. Surg..

[34] Doi J., Hirota J., Morita A., Fukushima K., Kamijyo H., Ohta H., Yamasaki M., Takahashi T., Katakura K., Oku Y. (2012). Intestinal Tritrichomonas suis (=*T. foetus*) infection in Japanese cats. J. Vet. Med. Sci..

[35] Lim S., Park S.I., Ahn K.S., Oh D.S., Ryu J.S., Shin S.S. (2010). First report of feline intestinal trichomoniasis caused by *Tritrichomonas foetus* in Korea. Korean J. Parasitol..

[36] Raab O., Greenwood S., Vanderstichel R., Gelens H. (2016). A cross-sectional study of *Tritrichomonas foetus* infection in feral and shelter cats in Prince Edward Island, Canada. Can. Vet. J..

[37] Hora A.S., Miyashiro S.I., Cassiano F.C., Brandão P.E., Reche-Junior A., Pena H.F.J. (2017). Report of the first clinical case of intestinal trichomoniasis caused by *Tritrichomonas foetus* in a cat with chronic diarrhoea in Brazil. BMC Vet. Res..

[38] Duarte R.P., Rocha P.R.D.A., Nakamura A.A., Cipriano R.S., Viol M.A., Melo G.D., Meireles M.V., Machado G.F. (2018). Detection of natural occurrence of *Tritrichomonas foetus* in cats in Araçatuba, São Paulo, Brazil. Pesqui. Veterinária Bras..

[39] Yao C., Köster L., Halper B., Dundas J., Nair R. (2018). Failure to detect *Tritrichomonas foetus* in a cross-sectional survey in the populations of feral cats and owned outpatient cats on St Kitts, West Indies. J. Feline Med. Surg. Open Rep..

[40] Veronesi F., Gazzonis A.L., Napoli E., Brianti E., Santoro A., Zanzani S.A., Olivieri E., Diaferia M., Giannetto S., Pennisi M.G. (2016). Cross-sectional survey on *Tritrichomonas foetus* infection in Italian cats. Vet. Parasitol. Reg. Stud. Rep..

[41] Xenoulis P.G., Saridomichelakis M.N., Read S.A., Suchodolski J.S., Steiner J.M. (2010). Detection of *Tritrichomonas foetus* in cats in Greece. J. Feline Med. Surg..

[42] Miró G., Hernández L., Montoya A., Arranz-Solís D., Dado D., Rojo-Montejo S., Mendoza-Ibarra J.A., Ortega-Mora L.M., Pedraza-Díaz S. (2011). First description of naturally acquired *Tritrichomonas foetus* infection in a Persian cattery in Spain. Parasitol. Res..

[43] Kuehner K.A., Marks S.L., Kass P.H., Sauter-Louis C., Grahn R.A., Barutzki D., Hartmann K. (2011). *Tritrichomonas foetus* infection in purebred cats in Germany: Prevalence of clinical signs and the role of co-infection with other enteroparasites. J. Feline Med. Surg..

[44] Tysnes K., Gjerde B., Nødtvedt A., Skancke E. (2011). A cross-sectional study of *Tritrichomonas foetus* infection among healthy cats at shows in Norway. Acta Vet. Scand..

[45] Profizi C., Cian A., Meloni D., Hugonnard M., Lambert V., Groud K., Gagnon A.C., Viscogliosi E., Zenner L. (2013). Prevalence of *Tritrichomonas foetus* infections in French catteries. Vet. Parasitol..

[46] Hedgespeth B.A., Stauffer S.H., Robertson J.B., Gookin J.L. (2020). Association of fecal sample collection technique and treatment history with *Tritrichomonas foetus* polymerase chain reaction test results in 1717 cats. J. Vet. Intern. Med..

[47] Crisi P.E., Paoletti B., Morelli S., Simonato G., Colombo M., Tiscar P.G., Boari A. (2021). *Tritrichomonas foetus* in cats from Central Italy: Clinical signs and risk factors. Vet. Parasitol. Reg. Stud. Rep..

[48] Gray S.G., Hunter S.A., Stone M.R., Gookin J.L. (2010). Assessment of reproductive tract disease in cats at risk for *Tritrichomonas foetus* infection. Am. J. Vet. Res..

[49] Arranz-Solís D., Pedraza-Díaz S., Miró G., Rojo-Montejo S., Hernández L., Ortega-Mora L.M., Collantes-Fernández E. (2016). *Tritrichomonas foetus* infection in cats with diarrhea from densely housed origins. Vet. Parasitol..

[50] Foster R.A., McGavin M.D., Zachary J.F. (2017). Pathologic Basis of Veterinary Disease.

[51] Manning K. (2010). Update on the diagnosis and management of *Tritrichomonas foetus* infections in cats. Top. Companion Anim. Med..

[52] Meggiolaro M.N., Roeber F., Kobylski V., Higgins D.P., Šlapeta J. (2019). Comparison of multiplexed-tandem real-time PCR panel with reference real-time PCR molecular diagnostic assays for detection of Giardia intestinalis and *Tritrichomonas foetus* in cats. Vet. Parasitol..

[53] Monteiro L., Bonnemaison D., Vekris A., Petry K.G., Bonnet J., Vidal R., Cabrita J., Mégraud F. (1997). Complex polysaccharides as PCR inhibitors in feces: Helicobacter pylori model. J. Clin. Microbiol..

[54] Schrader C., Schielke A., Ellerbroek L., Johne R. (2012). PCR inhibitors—occurrence, properties and removal. J. Appl. Microbiol..

[55] Stauffer S.H., Birkenheuer A.J., Levy M.G., Marr H., Gookin J.L. (2008). Evaluation of four DNA extraction methods for the detection of *Tritrichomonas foetus* in feline stool specimens by polymerase chain reaction. J. Vet. Diagn. Invest..

[56] Gookin J.L., Copple C.N., Papich M.G., Poore M.F., Stauffer S.H., Birkenheuer A.J., Twedt D.C., Levy M.G. (2006). Efficacy of ronidazole for treatment of feline *Tritrichomonas foetus* infection. J. Vet. Intern. Med..

[57] Act on the Protection of Animals Used for Scientific or Educational Purposes. https://isap.sejm.gov.pl/isap.nsf/DocDetails.xsp?id=WDU20150000266.

